# Enhanced Antibody Responses in a Novel NOG Transgenic Mouse with Restored Lymph Node Organogenesis

**DOI:** 10.3389/fimmu.2017.02017

**Published:** 2018-01-17

**Authors:** Takeshi Takahashi, Ikumi Katano, Ryoji Ito, Motohito Goto, Hayato Abe, Seiya Mizuno, Kenji Kawai, Fumihiro Sugiyama, Mamoru Ito

**Affiliations:** ^1^Central Institute for Experimental Animals, Kawasaki, Japan; ^2^Laboratory Animal Resource Center, University of Tsukuba, Tsukuba, Japan

**Keywords:** humanized mice, NOG, lymph node, T cell, homeostasis

## Abstract

Lymph nodes (LNs) are at the center of adaptive immune responses. Various exogenous substances are transported into LNs and a series of immune responses ensue after recognition by antigen–specific lymphocytes. Although humanized mice have been used to reconstitute the human immune system, most lack LNs due to deficiency of the interleukin (IL)-2Rγ gene (cytokine common γ chain, γc). In this study, we established a transgenic strain, NOG-pRORγt-γc, in the NOD/shi-*scid*-IL-2Rγ^null^ (NOG) background, in which the γc gene was expressed in a lymph-tissue inducer (LTi) lineage by the endogenous promoter of RORγt. In this strain, LN organogenesis was normalized and the number of human T cells substantially increased in the periphery after reconstitution of the human immune system by human hematopoietic stem cell transplantation. The distribution of human T cells differed between NOG-pRORγt-γc Tg and NOG-non Tg mice. About 40% of human T cells resided in LNs, primarily the mesenteric LNs. The LN-complemented humanized mice exhibited antigen-specific immunoglobulin G responses together and an increased number of IL-21^+^–producing CD4^+^ T cells in LNs. This novel mouse strain will facilitate recapitulation of human immune responses.

## Introduction

Reconstitution of the human immune system in immunodeficient mice enables investigation of human immunology and facilitates drug discovery (1–3). Progress in humanized mouse technology relies on extremely immunodeficient mouse strains; e.g., NOD*-scid* (4), NOD/Shi-*scid* IL2rγ*^null^* (NOG) (5), NOD/LtSz-*scid* IL2rγ*^null^* (NSG) (6), and BALB/c Rag2*^null^*IL2rγ*^null^* (BRG) (7). These platform strains are characterized by a severe deficiency in the murine immune system. In addition to deficiency of B and T lymphocytes due to *scid* gene mutation or disruption of the RAG-2 gene, especially, it is deletion of the interleukin (IL)-2 receptor γ (γc) gene that compromises the entire murine immune system. Because γc is a subunit for the receptors for six cytokines (IL-2, IL-4, IL-7, IL-9, IL-15, and IL-21) (8, 9), all biological pathways dependent on these cytokines are affected. In many cases, the primary consequences of the lack of γc are abnormal development and differentiation of lymphocytes; e.g., blocking of B-cell differentiation at the pre-proB cell stage (10), severe reduction in the number of T cells, and total loss of natural killer cells (11–13). There are also indirect secondary effects; e.g., impaired development of lymph nodes (LNs) in γc-deficient mice (11).

The organogenesis of LNs is complex and involves many cell types (14). One important cell type is the lymphoid tissue inducer (LTi) cell, which is a subpopulation in innate lymphoid cell 3 (15). During embryo development, LTi cells migrate toward lymphoid tissue stromal organizer (LTo) cells *via* a CXCL13-CXCR5–dependent mechanism (16–18). The critical molecule in the interaction between LTi and LTo cells is lymphotoxin (LT), which triggers LN formation (14). Differentiation of LTi cells requires expression of the master transcription factor, RORγt (19). IL-7 is necessary for their survival, as the number of LTi cells is reduced in γc-deficient mice; this reduction in numbers is responsible for the poor LN development (20). The transgenic expression of mouse thymic stromal lymphopoietin (TSLP), an IL-7 family molecule, restores the number of LTi in γc-deficient mice, and such TSLP transgenic (Tg) mice in a γc-deficient background showed normal LN development (20). These results suggest the importance of interactions between LTi cells and cytokines in LN organogenesis.

Because LNs are the primary sites of induction of immune responses; i.e., influx of antigen–loaded dendritic cells and subsequent activation of antigen-specific T- and B-cells resulting in germinal center formation, the absence of LNs could result in an immunodeficient status. Indeed, various mouse strains with no LNs—such as LTα^−/−^ mice (21), LTβ^−/−^ mice (22), or alymphoplasia mutant mice (*aly/aly*) (23), caused by a mutation in the NIK gene—show impaired or delayed immune responses. In addition, LNs are important for maintaining lymphocyte homeostasis (24).

Humanized NOG mice, which are produced by transplanting human CD34^+^ hematopoietic stem cells (HSCs), exhibit impaired LN development. In many cases, they have few small LNs even after full development of human B and T lymphocytes. Thus, it is plausible that the immune responses in humanized mice are insufficient due to their poor LN organogenesis. Indeed, such mice are deficient in antigen-specific responses, especially antigen-specific antibody responses (25–27).

In this study, we developed a novel NOG strain with LNs. We used a bacterial artificial chromosome (BAC) clone containing the entire RORγt locus in which the first exon of RORγt was replaced with the murine γc gene. The transgenic mice showed normal LN development in the NOG genetic background. After transplantation of human HSCs, these mice showed a significant increase in the total number of human T cells, body-wide redistribution of lymphocytes, and enhanced antibody production.

## Materials and Methods

### BAC Engineering

A BAC clone, RP23-263K17, containing the entire genomic region of the RORγ gene, was purchased from Advanced GenoTechs Co. (Tsukuba, Japan). BAC clone DNA was transfected into *Escherichia coli* EL250 by electroporation followed by homologous recombination (28). The whole cDNA of mouse γc and the polyA signal was introduced into the PL451 shuttle vector (28). The DNA fragment consisting of the murine γc and the neomycin resistance gene under the control of the PGK/EM7 promoter was amplified by Primestar GXL (Takara Bio Inc., Otsu, Japan). The PCR primer sequences are as follows: forward 5′-tgtgtgctgtcctgggctaccctactgaggaggacagggagccaagttctcagtcatgttgaaactattattgtcacc-3′, and reverse 5′-cctaggaatggtgacaggacccaggctcccccatgaccggatgcccccattcacttacgctctagaactagtggatcc-3′.

The PCR products were introduced into EL250 with RP23-263K17 to induce homologous recombination. After selecting chloramphenicol- and kanamycin-resistant colonies, we confirmed correct homologous recombination between the targeting vector and BAC DNA by sequencing and southern-blot analysis. The neomycin gene, which was flanked by flippase (FLP) recombinase target sequences, was removed by FLP-mediated site-specific recombination by arabinose treatment. As a result, the murine γc gene was inserted into exon 1 of the RORγt gene. BAC DNA was purified using NucleoBond BAC100 (Macherey-Nagel, Dueren, Germany).

### Mice and Reconstitution with Human Stem Cells

Mice were maintained in the animal facility at the Central Institute for Experimental Animals under specific-pathogen-free conditions. All animal experiments were approved by the Institutional Animal Care and Use Committee (certification number 11004A) and were conducted according to the institutional guidelines.

All of the experiments using human cells were approved by the Institutional Ethical Committee and conducted according to the guide lines.

Bacterial artificial chromosome transgenic B6 mice, which express murine γc under the control of RORγt regulatory elements, were generated in the C57/BL6 (B6) background. The BAC DNA described above was digested with PI-*Sce*I and purified. The linearized DNA was microinjected into B6 fertilized eggs by the standard protocol. The obtained mice were genotyped by PCR and a founder mouse was used for backcross mating. After seven-time backcross mating to the NOG strain, we confirmed the replacement of the genetic background from B6 to NOD using microsatellite markers. NOG-GM-CSF/IL-3 transgenic mice (NOG-GM3 Tg) were described elsewhere (29).

For reconstitution of the human immune system, 6-week-old male NOG or NOG-pRORγt-γc mice were irradiated with 180 cGy of X-rays (MBR-1520R-4, Hitachi, Hitachi, Japan) and 5 × 10^4^ umbilical cord blood CD34^+^ cells (StemExpress, Folsom, CA, USA) were transplanted by intravenous injection the next day (hereafter, hu-HSC-NOG or hu-HSC NOG-pRORγt-γc, respectively).

### Antibodies and Flow Cytometry

The following monoclonal antibodies (mAbs) were purchased from BioLegend (San Jose, CA, USA): anti-CD4-fluorescein isothiocyanate (FITC), anti-CD8a-FITC, anti-CD20-FITC, anti-CD33-FITC, anti-CD19-phycoerythrin (PE), anti-CD21-PE, anti-CD3-PECy7, anti-IgD-PECy7, anti-CD8a-allophycocyanin (APC), antimouse CD45-APC, anti-CD4-APCCy7, anti-CD19 APCCy7, and antihuman CD45-APCCy7.

To analyze human lymphocytes in mice reconstituted with the human immune system, multicolor cytometric analysis was performed using a fluorescence-activated cell sorter (FACS) Canto (BD Biosciences). Peripheral blood (PB) was collected from the retro-orbital venous plexus using heparinized pipettes periodically under anesthesia with isoflurane to monitor the development of human cells. PB was also assessed using a blood analyzer (XT-2000i, SYSMEX, Kobe, Japan) to enumerate total white blood cells. Red blood cells were eliminated using ACK solution (150 mM NH_4_Cl, 10 mM KHCO_3_, 1 mM EDTA-Na_2_) and mononuclear cells (MNCs) were stained with fluorescent marker-conjugated antibodies for flow cytometry.

At the time of euthanasia, MNCs were prepared from the thymus, spleen, LNs, or bone marrow (BM) by smashing with frosted slide glasses, or by flushing the femurs with FACS medium [phosphate-buffered saline (PBS) containing 2% fetal calf serum (FCS) with 0.1% NaN_3_] using a 27-gage needle. The cells were stained with the relevant mAb cocktails for 20 min on ice, and washed with cold FACS medium. The proportion of each lineage was calculated using FACS Diva software (BD Biosciences) and the absolute number of each fraction was determined by multiplying the frequency by the total cell number.

For intracellular staining, cells were suspended in Roswell Park Memorial Institute (RPMI) medium (RPMI + 2% FCS) and stimulated with phorbol myristate acetate (50 ng/ml) and ionomycin (1 µg/ml) in the presence of Brefeldin A (BioLegend) for 4 h at 37°C, then fixed with fixation buffer (eBioscience, San Diego, CA, USA). After permeabilization with Cytofix/Cytoperm solution (BD Biosciences), cells were stained with mAbs for anti-IFNγ-FITC, anti-IL-4-PE, and anti-IL-21-APC (BioLegend), together with antibodies for surface markers, for 20 min on ice. After the final wash, the cells were subjected to flow cytometry.

### Macroscopic Analysis of LNs

To visualize popliteal, inguinal, and sacral LNs, 1% Evans Blue dye (Sigma-Aldrich, St. Louis, MO, USA) was subcutaneously injected into the footpad or tail base. The mice were analyzed 1 h after injection; LNs were evidenced by accumulation of Evans Blue. In some cases, LNs were detected by stereoscopic microscopy.

### Immunohistochemistry

Mouse tissues were fixed in Mildform (Wako, Osaka, Japan), embedded in paraffin and sectioned using a microtome. We used a mouse antihuman CD3 (PS1, Nichirei, Tokyo, Japan) or anti-CD20 (L26, Leica Microsystems, Tokyo, Japan) antibody for human T or B cells, respectively. The specimens were stained using a Leica BOND-MAX automated immunohistochemistry stainer (Leica Microsystems, Tokyo, Japan).

### Enzyme-Linked Immunosorbent Assay (ELISA)

The total plasma human immunoglobulin (Ig) M and IgG levels in reconstituted NOG or NOG-pRORγt-γc mice were measured by ELISA using a human Ig assay kit (Bethyl, Denver, CO, USA).

To assay ovalbumin (OVA)-specific IgG antibodies, hu-HSC-NOG-GM-CSF/IL-3 Tg (NOG-GM3 Tg) or hu-HSC NOG-pRORγt-γc/GM-CSF/IL-3 Tg (NOG-pRORγt-γc/GM3 Tg) mice were immunized at 12 weeks following HSC transplantation three times every 10 days with mixture of 10 µg OVA (Sigma-Aldrich) with 2 mg Alum (Cosmo Bio, Tokyo, Japan) by intraperitoneal injection. Plasma from the immunized mice was harvested 4 days after the final immunization. Specific antibodies against OVA were measured by a standard method. Briefly, 96-well plates were coated with 5 µg/ml OVA at 4°C overnight. They were subsequently washed and blocked with PBS containing 1% bovine serum albumin. The collected plasma samples were loaded after threefold serial dilution to 1:6,561 in blocking solution. An HRP-conjugated antihuman Ig antibody was used as the secondary antibody. Anti-IgG- and -IgM-specific Abs were purchased from Bethyl. 3,3′,5,5′-Tetramethylbenzidine was used as a substrate for detection. The absorbance at 450 nm was measured using a microplate reader. The titer was defined as the dilution at which the absorbance of the sample became equivalent to that of non-immunized mice.

## Results

### Restoration of Mouse LN Organogenesis in NOG-pRORγt-γc Tg Mice

**Figure 1:**
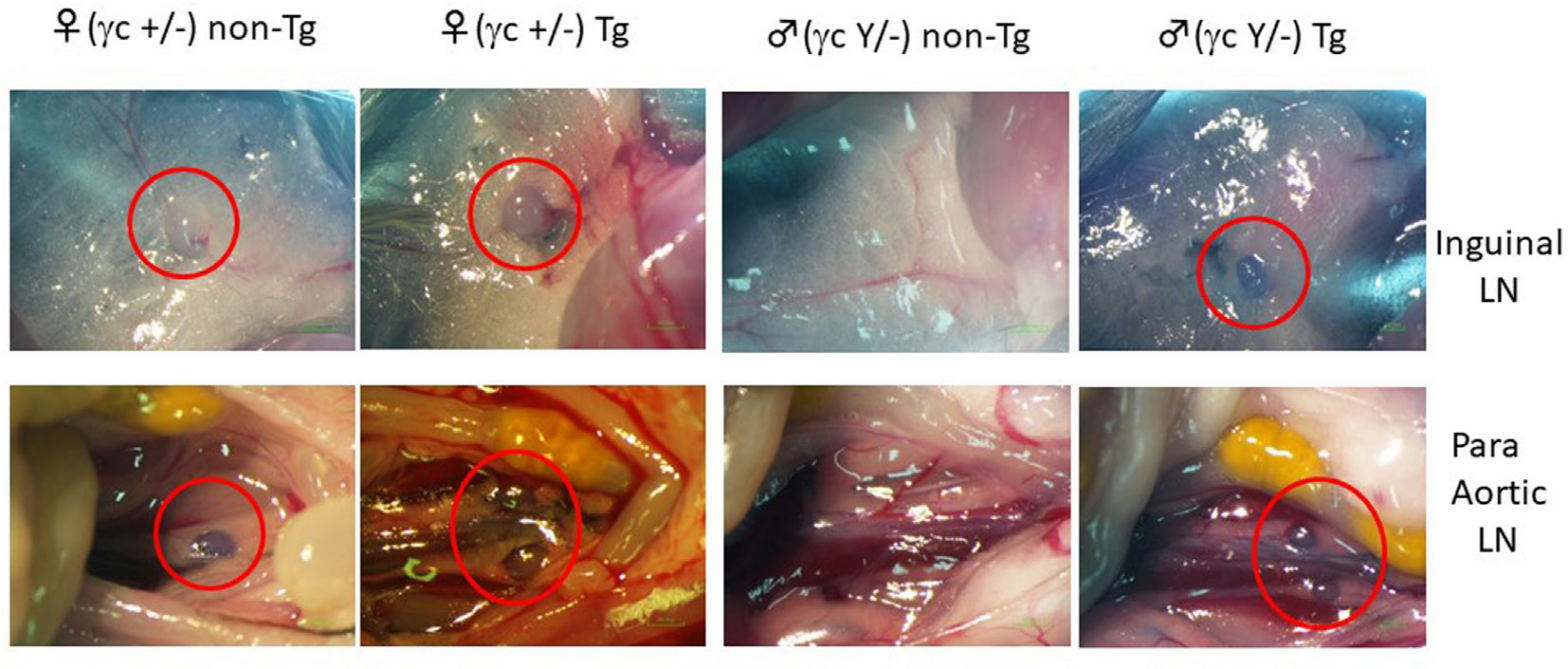
Induction of lymph node (LN) organogenesis in γc-deficient mice using the pRORγt-γc transgene. A bacterial artificial chromosome (BAC)-transgenic (Tg) male founder mouse was crossed with female NOD/shi-*scid*-IL-2Rγ^null^ (NOG) mice. The presence of LNs in the F1 mice was macroscopically confirmed. Red circles indicate inguinal LNs (top panels) and para-aortic LNs in the abdomen (bottom panels).

**Figure 2:**
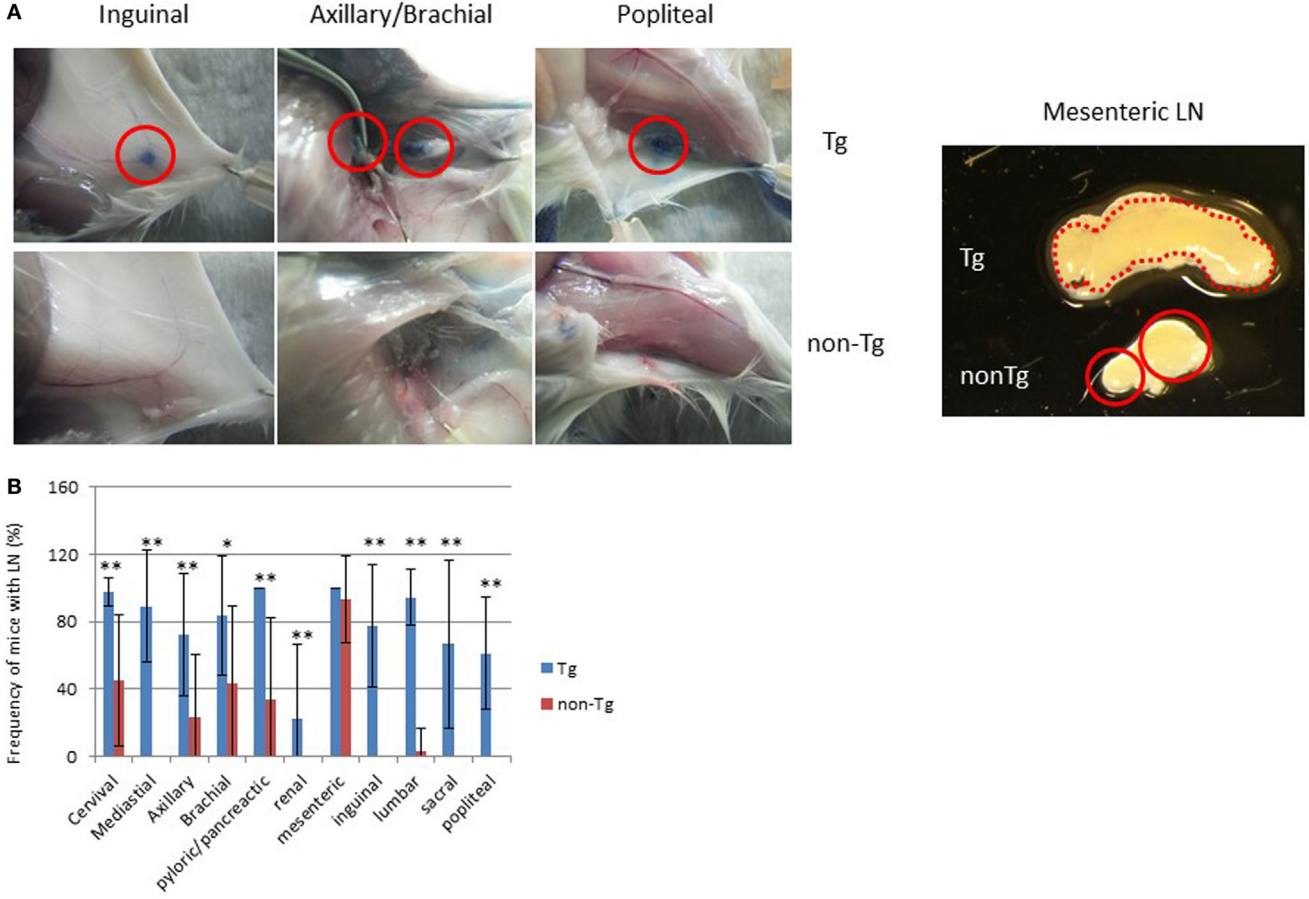
Restored lymph nodes (LNs) in NOG mice. **(A)** LNs in NOG-pRORγt-γc Tg and NOG non-Tg mice. Evans Blue was subcutaneously administered into the tail base or foot pad for visualization of draining LNs (left panels). Red circles indicate inguinal, axillary/brachial, and popliteal LNs. Representative mesenteric LNs (mLNs) from NOG-pRORγt-γc Tg and NOG non-Tg mice are shown in the right panel. Two distinct LNs from NOG mice are circled in red. **(B)** Efficiency of LN restoration in NOG–pRORγt-γc Tg mice. NOG-pRORγt-γc Tg or NOG non-Tg mice were examined for the presence of tissue-associated LNs. For scoring, the number of LNs in each tissue from individual mice was counted and the ratio to the number of the corresponding tissue-associated LNs in wild-type NOD mice was calculated. Mean ± SD from NOG-pRORγt-γc Tg (*n* = 9) and NOG non-Tg mice (*n* = 15). Student’s *t*-test was performed to assess statistical significance (*p < 0.05 and **p < 0.01).

To restore mouse LNs in the γc-deficient background, we attempted to express the mouse γc gene in an LTi-lineage-specific manner. Because RORγt is the critical master transcription factor for lineage specification, we generated a BAC transgenic strain in which expression of the γc gene was regulated by the endogenous control elements of the RORγt locus (Figure S1 in Supplementary Material). First, we investigated whether B6-pRORγt-γc Tg mice exhibited rescued normal LN development in the absence of the endogenous mouse γc gene. The Tg mice were crossed with γc-gene deficient mice (γc KO) to obtain the pRORγt-γc Tg in γc KO mice. Transgenic expression of the γc gene in the LTi-lineage restored LN development, which was absent in γc KO mice (Figure 1). After confirming the ability to stimulate LN organogenesis, we subsequently produced NOG-pRORγt-γc Tg mice (NOG-pRORγt-γc Tg) by backcrossing, and LN development in the NOG background was assessed (Figures 2A,B).

Macroscopic analysis revealed that most LNs were restored, although there were variances in the degree depending on the location. For example, restoration of cervical, mediastinal, and pyloric/pancreatic LNs was evident in almost 100% of NOG-pRORγt-γc Tg mice. The frequencies in NOG-non Tg mice of the same LNs were 30, 0, and 40%, respectively (Figure 2B). More than 80% of NOG-pRORγt-γc Tg mice had brachial, inguinal, and lumbar LNs, compared to 40, 0, and 5%, respectively, in NOG-non Tg mice. Axillary, sacral, and popliteal LNs were detected in about 50% of Tg mice, compared to 10, 0, and 0%, respectively, in NOG-non Tg mice. Renal LN development was not evident even in the Tg mice (Figure 2B). Another distinct feature of the Tg mice was enlargement of the mesenteric LNs (mLNs; Figure 2A). Whereas NOG-non Tg mice had two small distinct mLNs, Tg mice had a consecutive form of mLNs similar to those in WT mice (Figure 2A). However, we did not detect Peyer’s Patches (data not shown).

**Figure 3:**
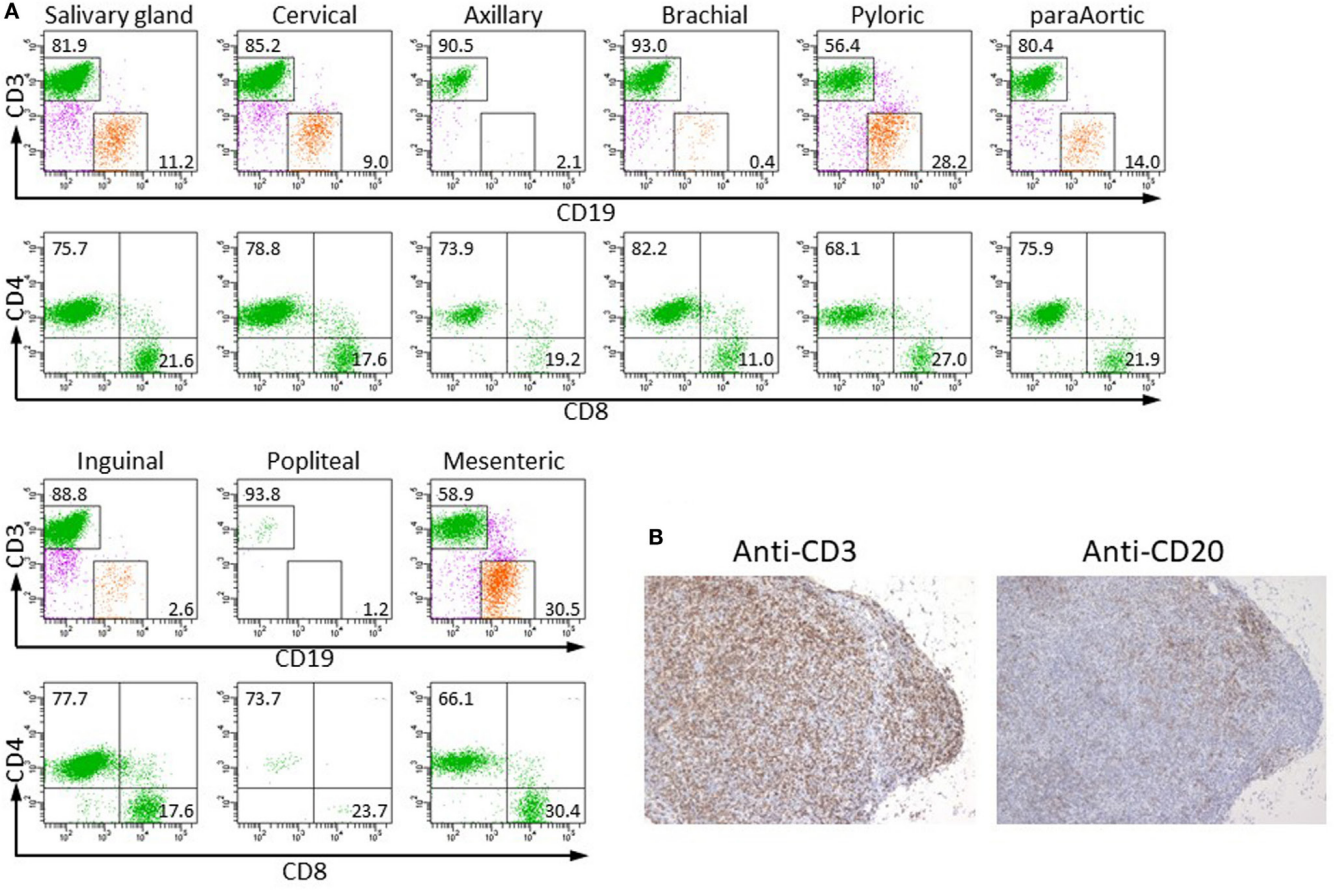
Colonization of restored lymph nodes (LNs) by human lymphocytes. **(A)** The indicated LNs were isolated from NOG-pRORγt-γc Tg mice at 20 weeks after hematopoietic stem cell transfer, and human lymphocytes were analyzed by fluorescence-activated cell sorting (FACS). A representative result from three different mice is shown. **(B)** Immunohistochemistry of mLNs. mLNs from the same mice used in **(A)** were fixed in formalin and sectioned after embedding in paraffin. Sections were stained with antihuman CD3 (left) and CD20 (right) antibodies.

To examine whether human lymphocytes could migrate and colonize the restored LNs of NOG-pRORγt-γc Tg mice, Tg mice were X-irradiated and transplanted with HSCs. After confirming development of human T cells in PB at 20 weeks post-HSC transplantation, we isolated LNs and analyzed the MNCs in the LNs by flow cytometry. Human lymphocytes were detected in all LNs. The subsets of human lymphocytes differed depending on the LN location. Although a considerable number of human B cells were detected in most of the LNs, the brachial, axillary, and popliteal LNs contained a few human B cells, 0–5% in human CD45^+^ cells. All LNs contained both human CD4^+^ and CD8^+^ T cells (Figure 3). Histological analysis of LNs showed a disorganized structure with a diffuse T-cell distribution rather than clear segregation of the B- and T-cell zones (Figure 3B). The disorganized structure was similar to that in NOG-non Tg mice (Figure S2 in Supplementary Material). We could not isolate a measurable number of human cells from the intestinal lamina propria in spite of the enlarged mLN in NOG-pRORγt-γc Tg mice (data not shown).

### Development of Human Lymphocytes in NOG-pRORγt-γc Tg Mice

**Figure 4:**
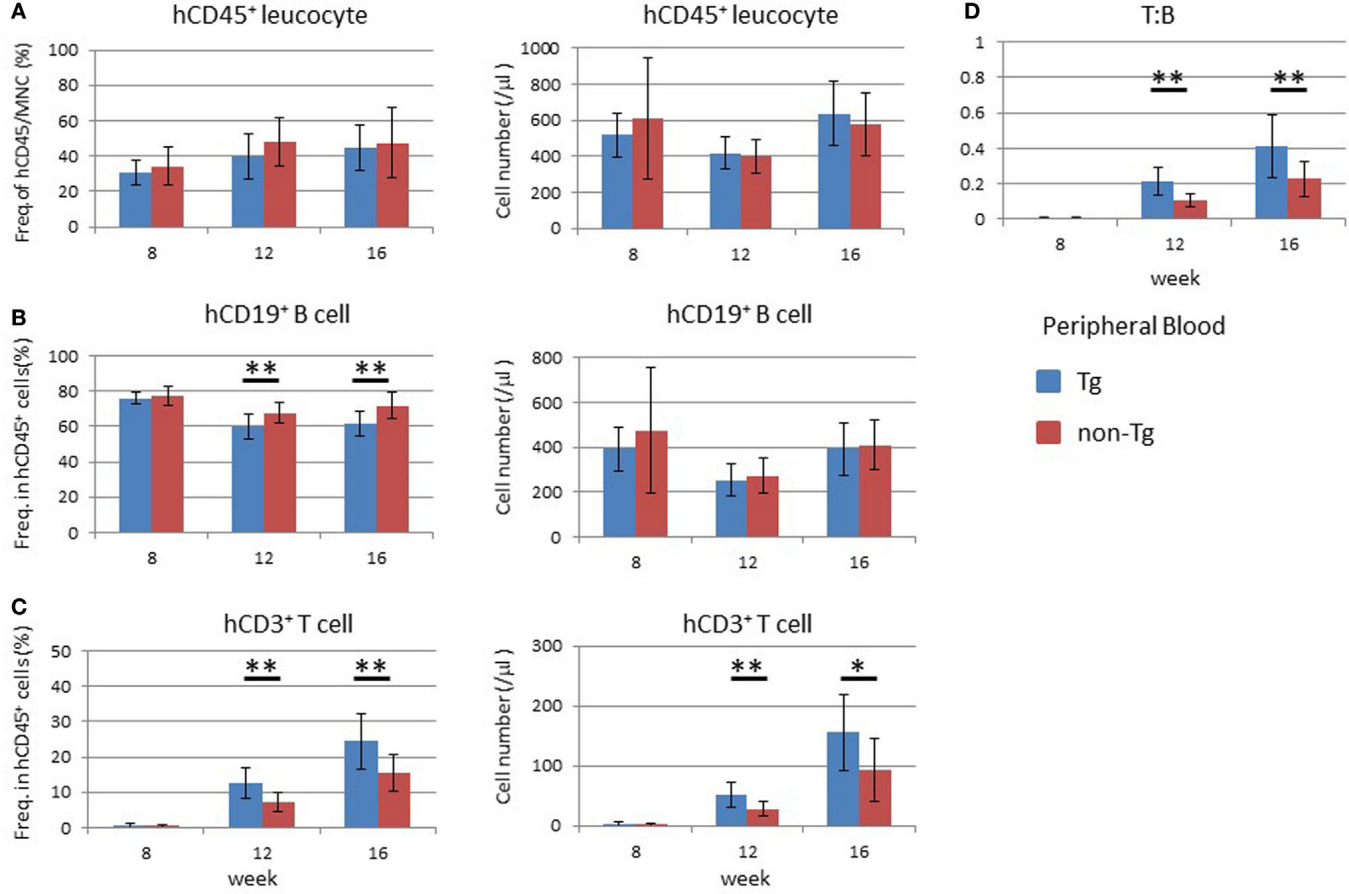
Development of human leukocytes in NOG-pRORγt-γc Tg mice. Peripheral blood (PB) was collected at the indicated time points after hematopoietic stem cell transfer. A portion was used for enumeration of total mononuclear cells. The remaining blood was subjected to fluorescence-activated cell sorter (FACS) for human leukocytes. **(A)** Frequency and absolute number of human CD45^+^ cells in total mononuclear cells. **(B,C)** Frequencies and absolute numbers of human B **(B)** and human T **(C)** cells among human CD45^+^ cells. Cellularity was calculated by multiplying the number of total mononuclear cells by the frequencies of each human subpopulation determined by FACS. **(D)** Kinetic change of the human T to B cell ratio. Mean ± SD from NOG-pRORγt-γc Tg (*n* = 12) and NOG non-Tg mice (*n* = 11). Student’s *t*-test was performed to assess statistical significance (*p < 0.05 and **p < 0.01). A representative result from three independent experiments is shown.

Human hematopoiesis was compared between NOG-non Tg and NOG-pRORγt-γc Tg mice. PB MNCs were analyzed 8–16 weeks after HSC transplantation (Figure 4). The frequency and number of human CD45^+^ cells did not differ between the two strains (Figure 4A). The development and differentiation of human CD33^+^ CD45^+^ myeloid cells were also comparable between non-Tg and pRORγt-γc Tg mice (data not shown). With respect to human lymphocytes, the development of human CD45^+^ leukocytes was not different at 8 weeks post-HSC transplantation. However, the frequency and absolute number of human T cells was significantly higher in pRORγt-γc Tg mice than in non-Tg mice at 12 weeks after HSC transplantation (Figure 4C). Although the frequency of human B cells was lower in Tg mice than in non-Tg mice, the absolute number of human B cells was not different (Figure 4B). Reflecting the increase in human T cells, the T:B ratio was higher in Tg mice than in non-Tg mice at 12 and 16 weeks after HSC transfer (Figure 4D).

**Figure 5:**
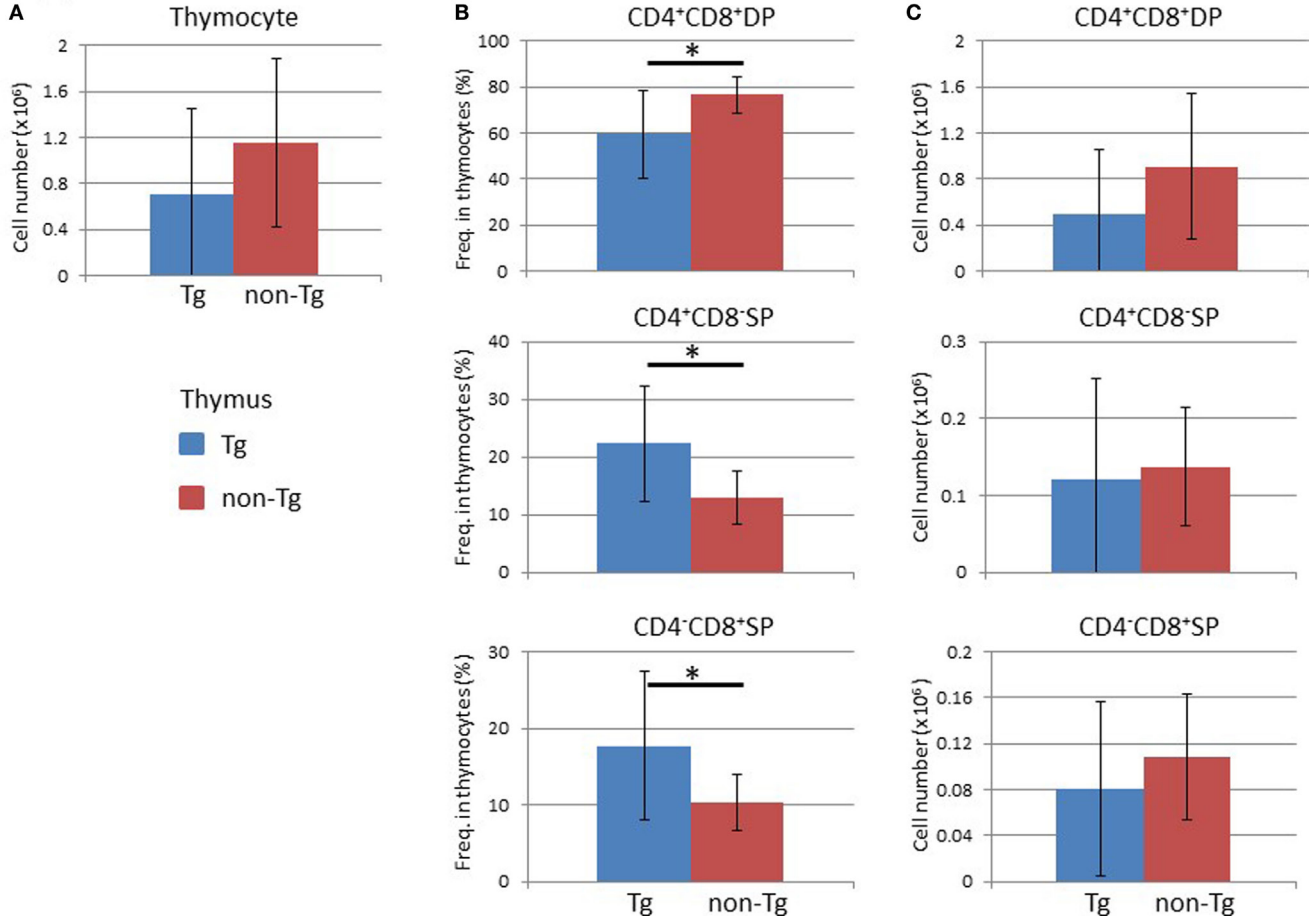
Analysis of human cells in the thymus. Thymocytes were prepared from the thymi of NOG-pRORγt-γc Tg and NOG non-Tg mice at 24 weeks after hematopoietic stem cell transplantation and analyzed by fluorescence-activated cell sorting. **(A)** Absolute numbers of human thymocytes. **(B,C)** The frequencies of human CD4^+^CD8^+^ double positive, CD4^+^CD8^−^, and CD4^−^CD8^+^ single positive cells in human thymocytes **(B)** and the absolute numbers of each cell subpopulation **(C)**. Mean ± SD of NOG-pRORγt-γc Tg (*n* = 12) and NOG non-Tg mice (*n* = 11). Asterisk indicates statistical significance (*p* < 0.05).

**Figure 6:**
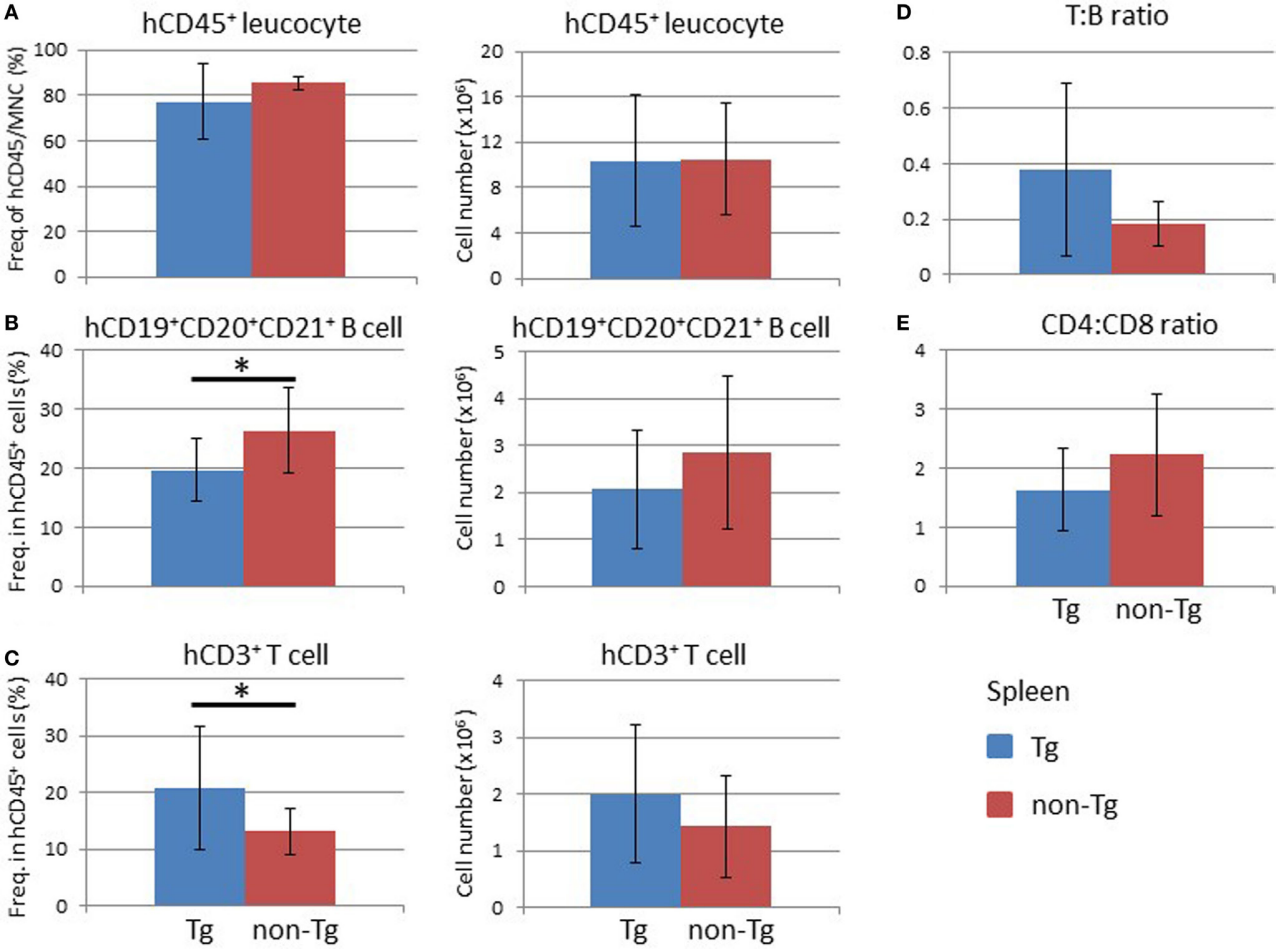
Analysis of human cells in the mouse spleen. Splenocytes were prepared from the spleens of NOG-pRORγt-γc Tg and NOG non-Tg mice for fluorescence-activated cell sorting analysis at 24 weeks after HSC transplantation. **(A)** Frequency and absolute number of human CD45^+^ cells in total mononuclear cells. **(B,C)** Frequencies and absolute numbers of human CD19^+^ B cells **(B)** and human CD3^+^ T cells **(C)** among human CD45^+^ cells. **(D)** Human T to B cell ratio in human CD45^+^ cells. **(E)** The CD4^+^ T cell to CD8^+^ T cell ratio in human T cells. Mean ± SD from NOG-pRORγt-γc Tg (*n* = 12) and NOG non-Tg mice (*n* = 11). Asterisk indicates statistical significance (*p < 0.05).

Analysis of the BM at 16 weeks after HSC transplantation demonstrated that the frequency and absolute number of human CD45^+^ leukocytes were higher in non-Tg mice than in Tg mice (Figure S3 in Supplementary Material). There were no significant differences in the frequencies and numbers of human CD19^+^ cells, which include human immature and mature B lineage cells, and human CD3^+^ T cells (Figure S3 in Supplementary Material). In the thymus, there was no significant difference in the cellularity of human thymocytes (Figure 5A). Analysis of subpopulations showed a significant reduction in the frequency of CD4^+^CD8^+^ thymocytes in NOG-pRORγt-γc Tg mice compared with non-Tg mice. In contrast, the frequencies of CD4^+^CD8^−^ and CD4^−^CD8^+^ thymocytes were higher in NOG-pRORγt-γc Tg mice than in NOG-non-Tg mice (Figure 5B). However, the absolute numbers of these subpopulations were not significantly different due to the large variances in the total number of thymocytes (Figure 5C). FACS analysis of splenocytes demonstrated that the frequency and absolute number of human CD45^+^ cells were not different irrespective of LN restoration (Figure 6A). A considerable portion of human CD19^+^ cells in hu-HSC NOG mice are immature B cells, including transitional B cells, and they do not express CD20 or CD21 (27). Thus, to examine the maturation of human B cells in NOG-pRORγt-γc Tg mice, we compared the frequency and number of the CD19^+^CD20^+^CD21^+^ subpopulation between NOG-pRORγt-γc Tg and NOG-non-Tg mice. While the frequency of mature human B cells was higher in non-Tg than in Tg mice, there was no significant difference in the absolute number (Figure 6B). In contrast, the frequency of human T cells was significantly higher in Tg than in non-Tg mice. We did not detect a significant difference in the cellularity of human T cells (Figure 6C). The human B to T cell ratio was comparable between Tg and non-Tg mice (Figure 6D). Regarding T cell subsets, the ratio of CD4^+^ to CD8^+^ T cells was not altered by the presence of LNs (Figure 6E).

**Figure 7:**
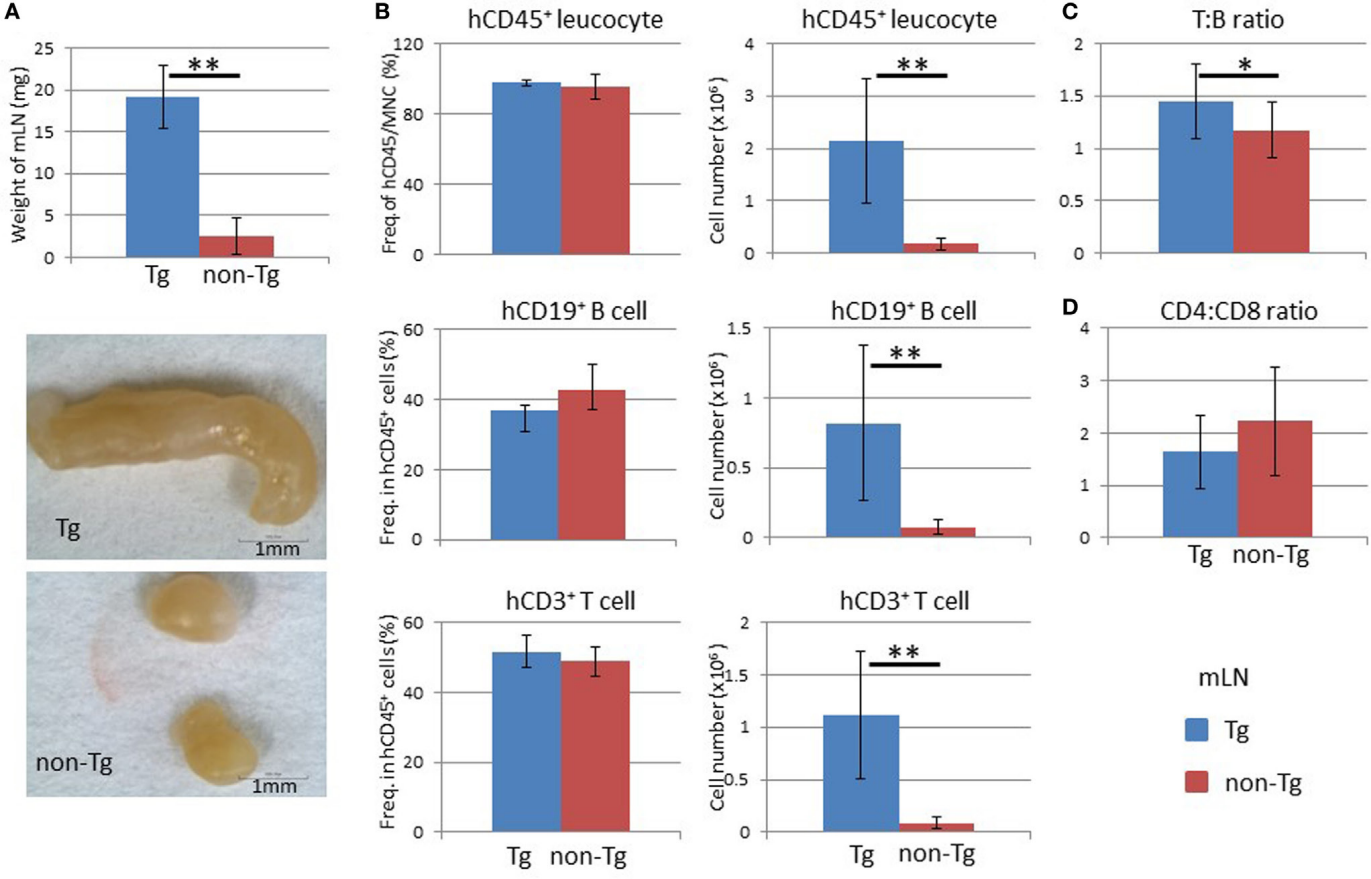
Analysis of mesenteric lymph nodes (mLNs). **(A)** Weight of mLNs. mLNs were isolated at 24 weeks after hematopoietic stem cell transplantation. Representative photographs of mLNs from human HSC-engrafted NOG-pRORγt-γc Tg and non-Tg mice. **(B)** Frequency and absolute number of human leukocytes. Human mononuclear cells were isolated from mLNs and analyzed by fluorescence-activated cell sorting. The number of human CD45^+^ leukocytes was obtained by multiplying the total number of mononuclear cells by the frequency of human CD45-positive cells. **(C)** Human T to B cell ratio. **(D)** Human CD4^+^ T cell to CD8^+^ T cell ratio. Mean ± SD from NOG-pRORγt-γc Tg (*n* = 12) and NOG non-Tg mice (*n* = 11). Student’s *t*-test was performed to assess statistical significance (*p < 0.05 and **p < 0.01).

We next examined LNs and found that pRORγt-γc Tg mice showed remarkable enlargement of mLNs. The weight of the mLNs in pRORγt-γc Tg mice was about eightfold higher than that in non-Tg mice (Figure 7A). Because other LNs were smaller than the mLNs, all LNs other than the mLNs were pooled for analysis; mLNs were analyzed separately. Reflecting the increase in weight, mLNs in NOG-pRORγt-γc Tg mice contained a significantly larger number of human leukocytes, which included both human CD19^+^ B cells and CD3^+^ T cells, than non-Tg mice (Figure 7B). The frequency of human CD45^+^ cells in total MNCs was not influenced, suggesting that mouse CD45^+^ cells were proportionally increased (Figure 7B). The T:B cell ratio was higher in Tg mice than in non-Tg mice (Figure 7C). As in the spleen, the CD4 to CD8 ratio was not different between Tg and non-Tg mice (Figure 7D).

**Figure 8:**
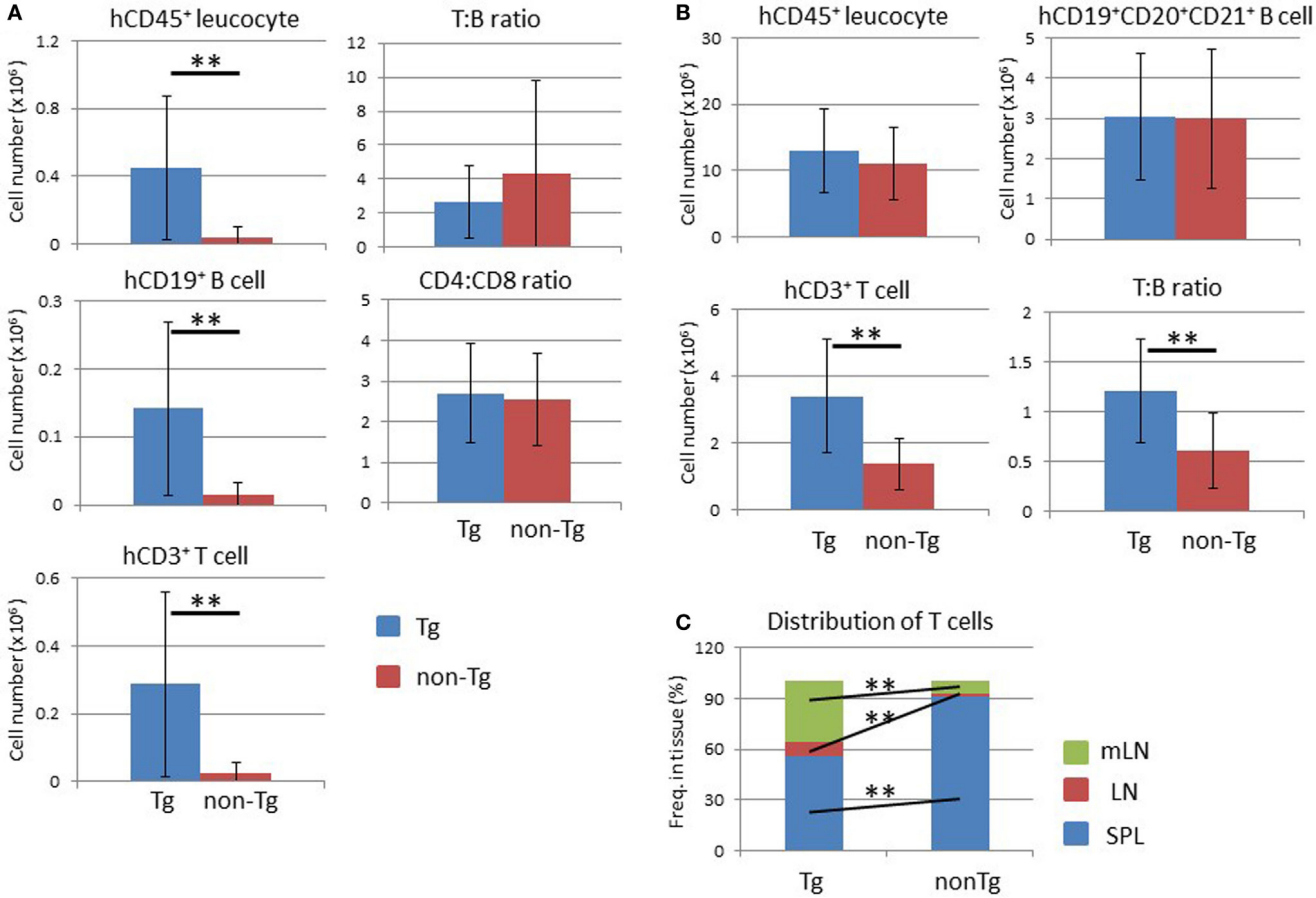
Analysis of lymph nodes (LNs) and distribution of human T lymphocytes. **(A)** Absolute number of human leukocytes in tissue-associated LNs. Human mononuclear cells were prepared from pooled LNs from NOG-pRORγt-γc Tg and NOG non-Tg mice at 24 weeks after hematopoietic stem cell transplantation. (Left panels) Numbers of human CD45^+^ leukocytes (upper), human B cells (middle), and human T cells (bottom) were calculated by multiplying the total number of mononuclear cells by the frequency of each fraction. (Right panels) The human T to B cell ratio (upper panel) and human CD4^+^ T cell to CD8^+^ T cell ratio (bottom panel). **(B)** Total number of human leukocytes in secondary lymphoid organs. Data from the spleen, LNs, and mesenteric LNs were summed for human CD45^+^ leukocytes (upper left), human B cells (upper right), and human T cells (bottom left). The human T to B cell ratio in all lymphoid organs is also shown (bottom right). **(C)** Distribution of human T cells. The proportion of human T cells in each secondary lymphoid organ was calculated. Mean ± SD from NOG-pRORγt-γc Tg (*n* = 12) and NOG non-Tg mice (*n* = 11). Statistical significance was evaluated by Student’s *t*-test (**p < 0.01).

An increased number of human leukocytes, including human CD19^+^ B and CD3^+^ T cells, in NOG-pRORγt-γc Tg mice was also observed in other tissue-associated LNs (Figure 8A). The frequency of human CD45^+^ cells was not influenced in tissue-associated LNs as in mLNs (data not shown). The ratio of these two populations remained unchanged between non-Tg and Tg mice (Figure 8A). The proportions of CD4^+^ and CD8^+^ T cells in CD3^+^ T cells also did not differ between non-Tg and Tg mice (Figure 8A).

After determining the absolute number of human lymphocytes in secondary lymphoid organs (spleen, LNs, and mLNs), the total number of human cells in the whole mouse was calculated. There was no significant difference in the human CD45^+^ cell number between non-Tg and Tg mice (Figure 8B). Interestingly, the total number of human CD3^+^ T cells increased about threefold in Tg mice compared to non-Tg mice (Figure 8B), while the number of human CD19^+^CD20^+^CD21^+^ mature B cells was not significantly different (Figure 8B). Accordingly, the T to B cell ratio was higher in NOG-pRORγt-γc Tg mice than in NOG-non-Tg mice. Due to the migration of human lymphocytes into LNs, the lymphocyte tissue distribution differed markedly between NOG-pRORγt-γc Tg mice and non-Tg mice. In normal NOG non-Tg mice, almost 90% of human T cells resided in the spleen. In contrast, ≤60% of human T cells were present in the spleen in Tg mice, and ~30 and 10% of human T cells migrated into mLNs or other tissue-associated LNs, respectively (Figure 8C). Mature human B cells were also distributed primarily in LNs (data not shown).

### Augmentation of Humoral Immune Responses in NOG-pRORγt-γc Tg Mice

**Figure 9:**
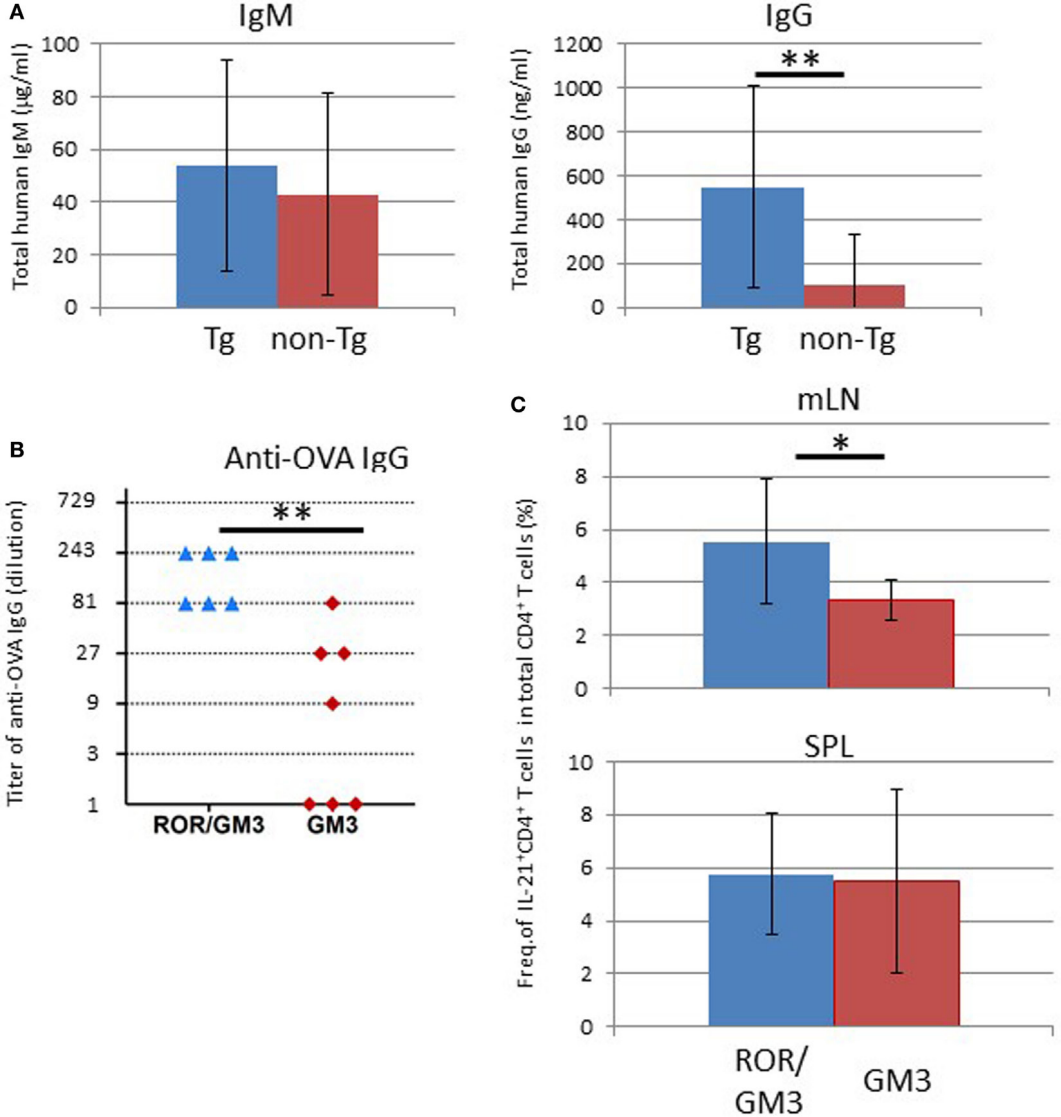
Enhanced humoral responses in NOG-pRORγt-γc Tg mice. **(A)** Total IgM and IgG levels in plasma. Plasma was prepared 24 weeks after hematopoietic stem cell (HSC) transplantation and total IgM and IgG levels were quantified by enzyme-linked immunosorbent assay (ELISA). Mean ± SD from NOG-pRORγt-γc Tg (*n* = 12) and NOG non-Tg mice (*n* = 11). Statistical significance was evaluated by Student’s *t*-test (**p < 0.01). **(B)** ELISA results for ovalbumin (OVA)-specific human IgG. NOG-pRORγt-γc/GM-CSF/IL-3 Tg (ROR/GM3) (*n* = 6) or NOG-GM-CSF/IL-3 Tg (GM3) (*n* = 7) mice were transplanted with HSCs. After confirming T-cell differentiation in the peripheral blood at 12 weeks post-HSC engraftment, mice were immunized three times with OVA/Alum. Plasma was collected 4 days after the final boost. Titers of individual mice are shown. Statistical significance was evaluated by Student’s *t*-test (**p < 0.01). **(C)** Increase in the number of IL-21-producing CD4^+^ T cells in lymph nodes (LNs). Spleen and mesenteric LNs from OVA-immunized NOG-ROR/GM3 or NOG-GM3 mice were stimulated with phorbol myristate acetate/ionomycin for 4 h in the presence of Brefeldin A. Cytokines were stained after fixation and permeabilization. Mean ± SD frequencies of IL-21^+^ CD4^+^ T cells among total CD4^+^ T cells. Statistical significance was evaluated by Student’s *t*-test (*p < 0.05).

To examine the immunological features of hu-HSC NOG-pRORγt-γc Tg mice, serum total human IgM and IgG levels were quantified by ELISA. The IgM level was equivalent in non-Tg and Tg mice, whereas the IgG level was significantly higher in Tg mice than in non-Tg mice (Figure 9A). Next, we investigated whether LN-sufficient humanized mice could induce antigen-specific humoral immune responses. Impaired production of antigen-specific IgG responses in humanized mice has been reported, likely due to the lack of cognate interactions between mouse major histocompatibility complex (MHC)-restricted human T cells and human leukocyte antigen (HLA) on human B cells (30, 31). However, antigen-specific IgG responses could be facilitated by crosstalk between antigen-specific B and T cells in LNs. To further improve the probability of human immune responses, we used transgenic mice the GM-CSF/IL-3 transgenic NOG (NOG-GM3 Tg). This strain allowed the development of various lineages of human cells, including lymphoid and myeloid cells, from HSCs (29). Those human cells could facilitate induction of immune responses. After reconstitution of NOG-pRORγt-γc/GM3 Tg or NOG-GM3 Tg mice with the human immune system, we immunized the animals with OVA/Alum complex. The OVA-specific human IgG titer was significantly higher in NOG-pRORγt-γc/GM3 Tg than in NOG-GM3 Tg mice (Figure 9B), and was ~250-fold higher than that in non-immunized mice. Although we expected that improved human hematopoiesis enhanced antigen-specific antibody responses in NOG-GM3 Tg mice, the induction of OVA-specific IgG was modest. Next, we investigated the production of IL-4 and IL-21 by CD4^+^ T cells, because these cytokines are important for promoting class switching and plasma cell differentiation (32, 33). The frequency of IL-21^+^ CD4^+^ T cells was significantly higher in mLNs from NOG-pRORγt-γc/GM3 Tg than NOG-GM3 Tg mice (Figure 9C), but this was not the case in splenic CD4^+^ T cells from the same animals. There were no differences in the frequency of IFNγ-, IFN-17-, and IL-4–producing CD4^+^ T cells between NOG-pRORγt-γc/GM3 Tg and NOG-GM3 Tg mice (Figure S4 in Supplementary Material). A standard immunophenotyping protocol using chemokine receptor expression also confirmed the increase of CD3^+^CD4^+^CD5RA^−^CXCR5^+^ follicular helper T cells (Tfh) in frequency (34) and there was no difference in the frequency of FOXP3^+^ CXCR4^+^CD25^+^ CD4^+^ human regulatory T cells (Treg) in CD4^+^ T cells between NOG-pRORγt-γc Tg and non-Tg mice (Figure S5 in Supplementary Material) (35). These results suggest that the composition of T cell subsets was generally maintained in NOG-pRORγt-γc Tg mice except the increase of IL-21^+^ producing Tfh cells. It should be noted, however, that the absolute cell number of each subset significantly increased reflecting the increase of total CD4^+^ T cells.

## Discussion

In this study, we demonstrated that LN organogenesis could be restored in NOG mice by expressing the mouse γc gene under the control of the RORγt promoter, and that these LNs function as a reservoir for human lymphocytes after reconstitution of the human immune system. Furthermore, our results showed that restored LNs could confer immunological competence on humanized NOG mice.

To restore LN development in NOG mice, we generated a NOG transgenic strain expressing human TSLP. This approach was not successful, however, in the NOG background, as our NOG transgenic strain expressing the human TSLP gene, which possesses about 42% homology with the mouse TSLP gene, developed severe thymoma, which resembled the disease frequently seen in NOD-*scid* mice (data not shown) (36). It is possible that cytokine signaling through mouse IL-7Rα or mouse TSLP receptor stimulated oncogenic mechanisms intrinsic to mice with the NOD background. The efficiency of LN restoration was greater in TSLP Tg γc-KO mice than in NOG-pRORγt-γc Tg mice. Indeed, some NOG-pRORγt-γc Tg mice showed unilateral development of axillary, brachial, inguinal, or popliteal LNs, while the TSLP Tg γc-KO mice showed almost 100% LN organogenesis (20). It is possible that the expression level of γc in LTi cells was not sufficient for full recovery of this lineage, resulting in partial development of LNs in NOG-pRORγt-γc Tg mice. Supporting this hypothesis, although we detected significant increase of the frequency and number of LTi cells in NOG-pRORγt-γc Tg mice compared with NOG-non-Tg mice. However, the increase was not more than twofold in number (Figure S6 in Supplementary Material). This may also explain the lack of Peyer’s patches. Mice with constitutive expression of the mouse γc gene with strong promoters could results in the better restoration of LN development and Peyer’s patches. As a result, such strains, an equivalent strain to NOD-*scid* mice, may have better organized LN structures and elicit better immune responses like in NOD-*scid* mice (37). However, at the expense of the benefit, such strains may develop thymoma (36). In addition, they may have various lymphoid lineages which reduce the efficiency of engraftment of human hematopoietic cells (38).

Humanized mice generated by simple transfer of human cord blood-derived HSCs exhibit suboptimal immune responses (1). These weak immune reactions can be in part explained by inefficient development of human T cells by the atrophic mouse thymus, lack of HLA-restriction of human T cells (27), incomplete maturation of human B cells (27), or accumulation of human T cells susceptible to cell death by antigen stimulation (27). These problems have been addressed by various approaches. For example, administration of recombinant human Fc-IL-7 protein (39) or lentiviral delivery of human IL-7 increased T-cell numbers (40). HLA-matching between HSC donor and recipient mice by introducing HLA transgenes into mice induced HLA-restricted human immune responses (30, 31, 41, 42). As an alternative approach to overcome the limitations inherent to humanized mice, we examined whether restoration of LNs would improve human lymphocyte homeostasis and human adaptive immune responses.

Preferential expansion of human T cells was evident in NOG-pRORγt-γc Tg mice. In addition, restoration of LNs induced a significant redistribution of human lymphocytes from the spleen to the LNs. Indeed, almost 40% of total human T cells were mobilized into LNs in NOG-pRORγt-γc Tg mice, mostly to the mLNs. The macroscopic analysis demonstrated that the weight of the mLNs in NOG-pRORγt-γc Tg mice was eightfold higher than that in NOG non-Tg mice, and they harbored ~35% of the total human T cells. Although the reason for the preferred residence of human T cells in mLNs is unclear, not only simple migration but also homeostatic proliferation may be strongly induced in the enlarged mLNs in NOG–pRORγt-γc Tg mice. This seems to be mediated by thymus-independent mechanisms, as the numbers of thymocytes were not different between Tg and non-Tg mice. By draining the small intestine and colon, mLNs provide human T cells with abundant and non-competitive signals, which include pMHC, cytokines, and physical space. Thus, mLNs may have a marked impact on the homeostasis of human T cells. In contrast to the increase in the number of human T cells, the effect on human B cells was unremarkable. The absolute number of human mature B cells was not significantly different between NOG-pRORγt-γc Tg and NOG non-Tg mice. Although the development of human T cells and the maturation of human B cells is reportedly correlated in humanized mice, the robust increase in human T cell number did not result in an increase in that of human mature B cells in NOG-pRORγt-γc Tg mice (43). Furthermore, histological analysis showed incomplete architecture of LNs, which lacked B-cell follicles and germinal centers. The persistent blockage of B cell maturation suggests that LNs do not provide an environment conducive to full maturation of human B cells. Considering the abundant T cells in LNs, T cell-independent factors may be necessary for B cell maturation. Our immunohistochemistry showed that mouse follicular dendritic cells (FDCs) were not induced in mLNs (data not shown). This may be due to the absence of interaction between mature human B cells and mouse FDC progenitor cells. Alternatively, human FDCs, which were of non-hematopoietic origin, may be necessary for inducing maturation of human B cells and thus organizing LN structures.

The serological analysis demonstrated induction of a partial human humoral immune response in the LN-sufficient humanized mice, despite the lack of HLA-II molecules. Introduction of HLA-DR molecules in recipient mice and matching of the HLA-DR haplotype between the recipient mice and HSC donors are essential for induction of antigen-specific IgG responses in humanized mice (30, 31). The importance of HLA-II in recipient mice was also confirmed in this study using NOG-GM/3 Tg mice, which did not show antigen-specific IgG responses despite differentiation of multiple types of human antigen-presenting cells, including dendritic cells, macrophages/monocytes, and B cells, in the spleen. Although the mechanisms for the induction of antigen-specific IgG responses in LN-restored humanized mice are unclear, the increases in the frequency of IL-21 producing CD4^+^ T cells and the total number of CD4^+^ T cells in LNs suggest that an IL-21-rich milieu is generated in LNs, which induces IgG class switch recombination in B cells in the vicinity, even in the absence of cognate interactions with T cells with the same antigen specificity. Introduction of HLA-II molecules may further enhance antibody production.

In this study, we developed a novel NOG substrain with immunologically competent LNs. The enhanced immune responses of NOG-pRORγt-γc Tg mice will be useful, particularly in combination with HLA Tg or human cytokine-gene introduced mouse strains. This would synergistically enhance the quasihuman immune response and facilitate development of novel vaccines against infectious diseases and immunotherapies for tumors.

## Author Contributions

TT designed the study, performed the data analysis, and wrote the manuscript. IK and TT conducted all of the experiments. MG, SM, and FS performed the embryo manipulation. HA maintained the NOG-pROPγt-γc Tg strains. KK was responsible for the pathological analysis. MI, RI, and TT organized the project.

## Conflict of Interest Statement

The authors declare that the research was conducted in the absence of any commercial or financial relationships that could be construed as a potential conflict of interest.

## Supplementary Material

Figure S1 Schematic of bacterial artificial chromosome recombination for expression of mouse interleukin 2Rγ under the control of the regulatory elements of mouse RORγt.

Figure S2 Immunohistochemistry of mesenteric lymph node in hu-HSC NOG-non Tg mice.

Figure S3 Analysis of human cells in the bone marrow (BM). BM from NOG-pRORγt-γc Tg and NOG non-Tg mice were isolated from the tibiae and analyzed by fluorescence-activated cell sorting (FACS). **(A)** Frequency and absolute number of human CD45^+^ cells among total mononuclear cells. **(B,C)** Frequencies and absolute numbers of human CD19^+^ B cells **(B)** and human CD3^+^ T cells **(C)** among human CD45^+^ cells. Mean ± SD from NOG-pRORγt-γc Tg (*n* = 12) and NOG non-Tg mice (*n* = 11).

Figure S4 Frequency of human Th1, Th2, and Th17 cells in hu-HSC NOG-pRORγt-γc Tg. The mesenteric lymph node cells used in Figure 9 were stained for human IFN-γ (Th1 cells), IL-4 (Th2 cells), and IL-17 (Th17 cells).

Figure S5 Subpopulation in CD4^+^ T cells. Spleen and mesenteric lymph node in NOG-pRORγt-γc Tg and NOG-non-Tg mice were analyzed at 16weeks after hematopoietic stem cell transplantation (*n* = 4). Th1 cells, Th17, or Tfh cells were defined as CD3^+^CD4^+^CD45RA^−^CXCR5^−^CXCR3^+^CCR6^−^, CD3^+^CD4^+^CD45RA^−^CXCR5^−^CXCR3^−^CCR6^+^, or CD3^+^CD4^+^CD45RA^−^CXCR5^−^ cells. Human regulatory T cells were defined as FOXP3^+^CCR4^+^CD25^+^CD4^+^ T cells. For immunophenotyping, following antibodies were used for staining and analyzed by a BD LSR Fortessa X-20 cell analyzer (BD Biosciences). Anti-CCR7-Brilliant Violet 421, anti-CD45-BV510, anti-CXCR3-APC, anti-CD4-APC, anti-CD45RA-APCCy7, anti-CCR6-PE, anti-CD4-PECy7, anti-CCR4-PECy7, and antimouse CD45-PerCP-Cy5.5 were from BioLegend. Anti-CXCR5-Brilliant Blue 515, anti-CD25-BB515, and anti-CD3-Brilliant Ultraviolet 737 were from BD Biosciences. Dead cells were excluded by 7-AAD (Beckman Coulter). Intracellular staining of FOXP3 was conducted using Anti-Human Foxp3 staining Set phycoerythrin from eBioscience according to the manufacturer’s instruction. Student’s *t*-test was performed to assess statistical significance (**p* < 0.05).

Figure S6 Increase of LTi cells in NOG-pRORγt-γc Tg. The presence of LTi was examined in embryo of NOG-pRORγt-γc Tg and NOG-non-Tg mice at E15. The fetal intestine was smashed with a pestle and mononuclear cells were stained with a cocktail of antibodies; antimouse CD3-FITC, antimouse CD4-PE, antimouse B220, antimouse CD127 (IL-7Rα)-APC, and antimouse CD45-APC-Cy7. LTi cells were defined as CD4^+^CD127^+^ cells in CD45^+^CD3^−^B220^−^ cells. A part of embryo was used for genotyping by PCR. Mean ± SD from NOG-pRORγt-γc Tg (*n* = 12) and NOG non-Tg mice (*n* = 14). Student’s *t*-test was performed to assess statistical significance (**p* < 0.05).
