# Supplementary figures and images for: Enhanced Antibody Responses in a Novel NOG Transgenic Mouse with Restored Lymph Node Organogenesis
Source: Front Immunol. 2018 Jan 17;8:2017. doi: 10.3389/fimmu.2017.02017 (PMC5776085; doi:10.3389/fimmu.2017.02017)

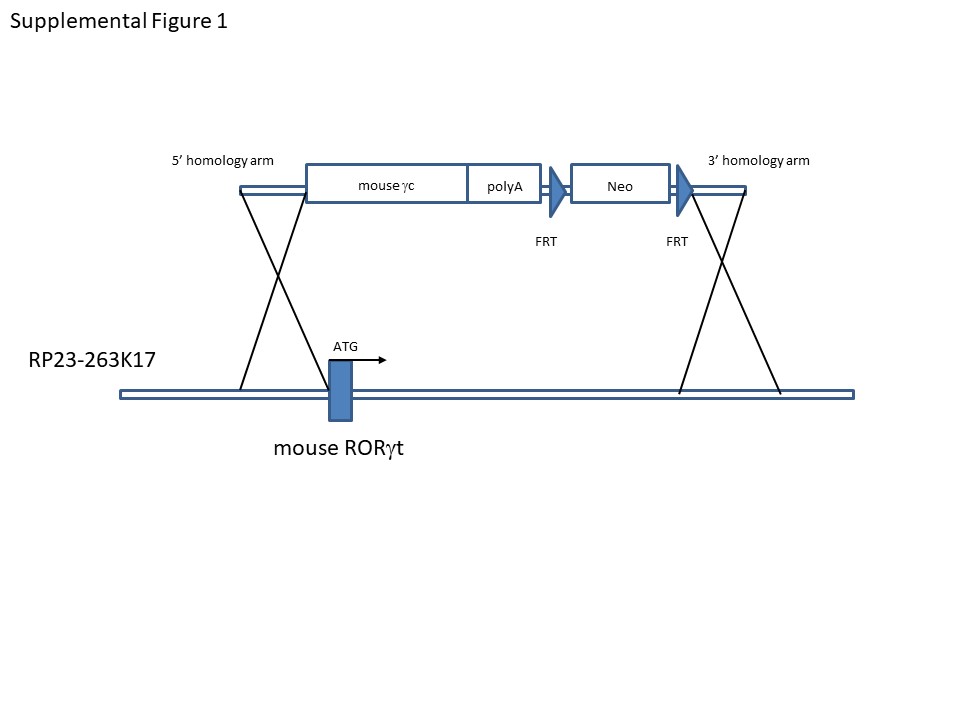

Supplement: Figure S1 — Schematic of bacterial artificial chromosome recombination for expression of mouse interleukin 2Rγ under the control of the regulatory elements of mouse RORγt. [file image_1.jpeg]

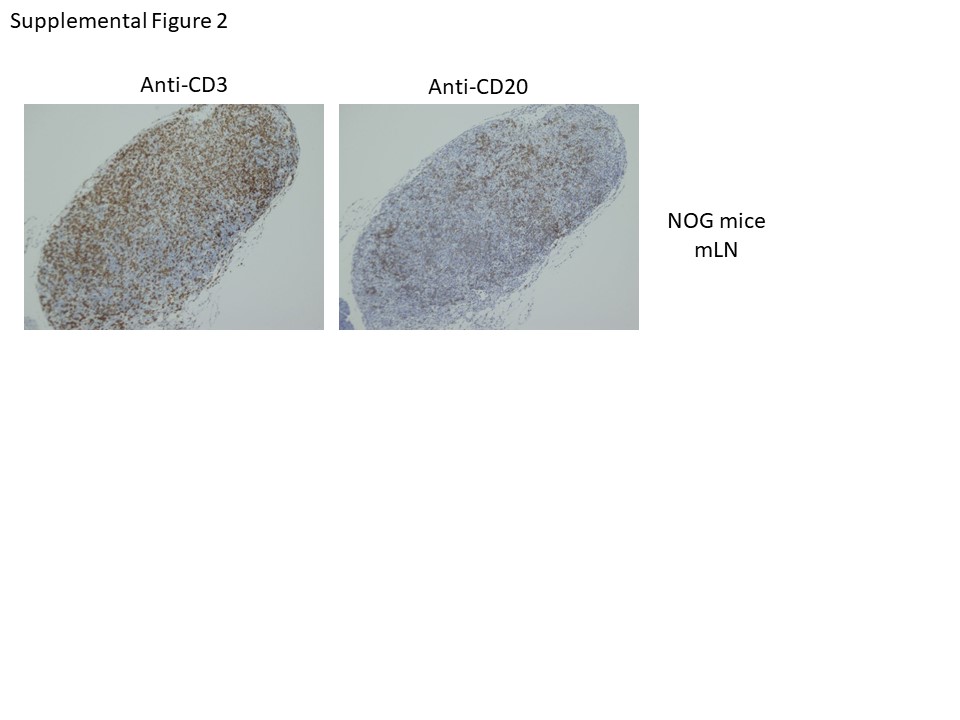

Supplement: Figure S2 — Immunohistochemistry of mesenteric lymph node in hu-HSC NOG-non Tg mice. [file image_2.jpeg]

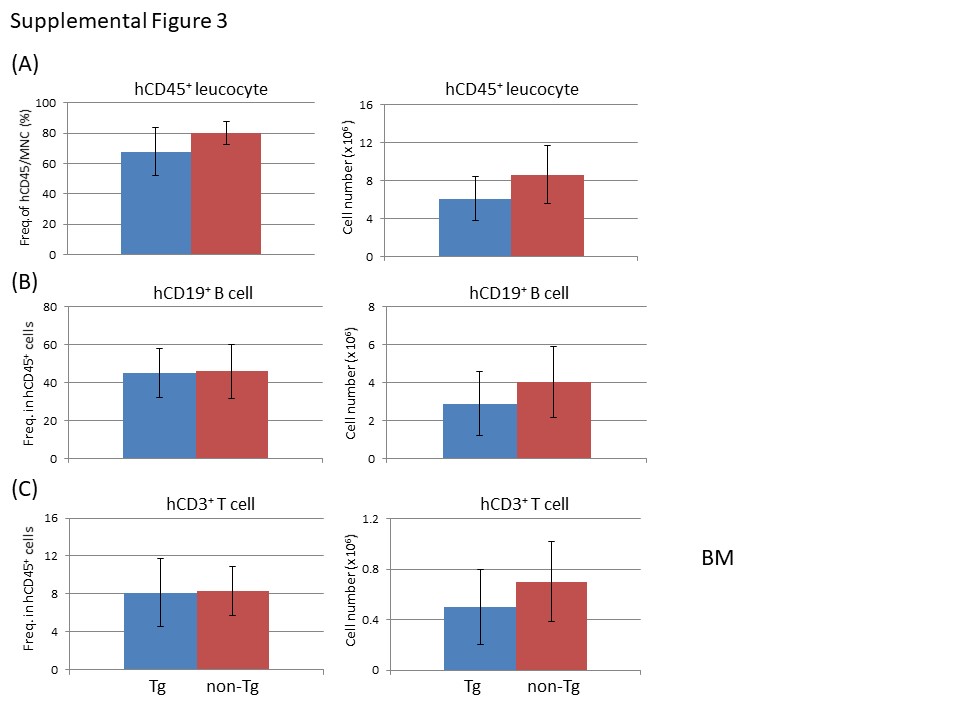

Supplement: Figure S3 — Analysis of human cells in the bone marrow (BM). BM from NOG-pRORγt-γc Tg and NOG non-Tg mice were isolated from the tibiae and analyzed by fluorescence-activated cell sorting (FACS). (A) Frequency and absolute number of human CD45+ cells among total mononuclear cells. (B,C) Frequencies and absolute numbers of human CD19+ B cells (B) and human CD3+ T cells (C) among human CD45+ cells. Mean ± SD from NOG-pRORγt-γc Tg (n = 12) and NOG non-Tg mice (n = 11). [file image_3.jpeg]

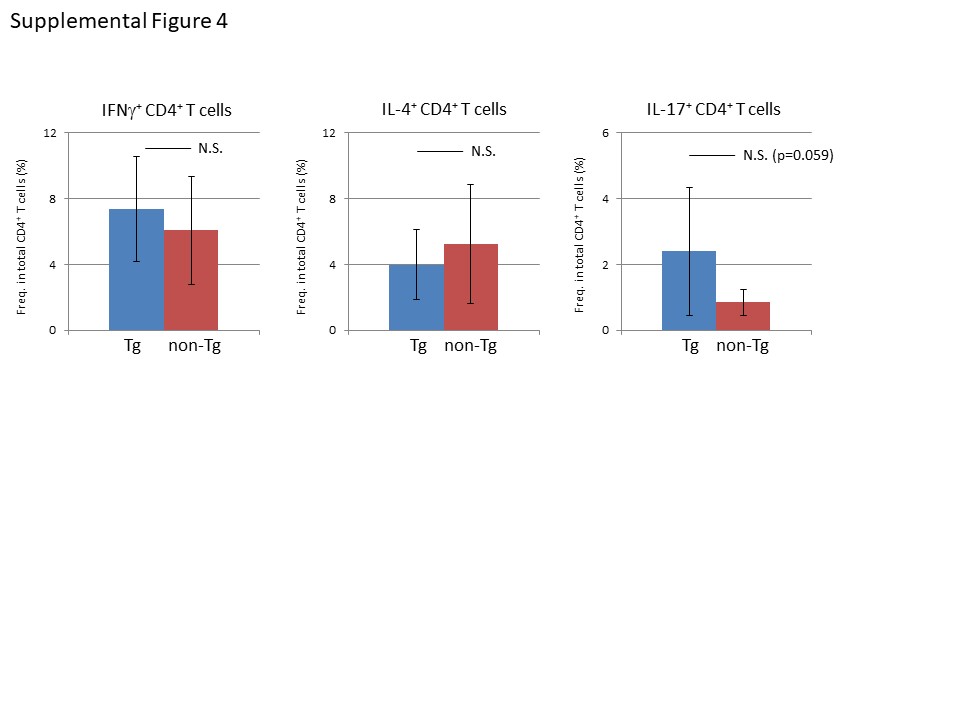

Supplement: Figure S4 — Frequency of human Th1, Th2, and Th17 cells in hu-HSC NOG-pRORγt-γc Tg. The mesenteric lymph node cells used in Figure 9 were stained for human IFN-γ (Th1 cells), IL-4 (Th2 cells), and IL-17 (Th17 cells). [file image_4.jpeg]

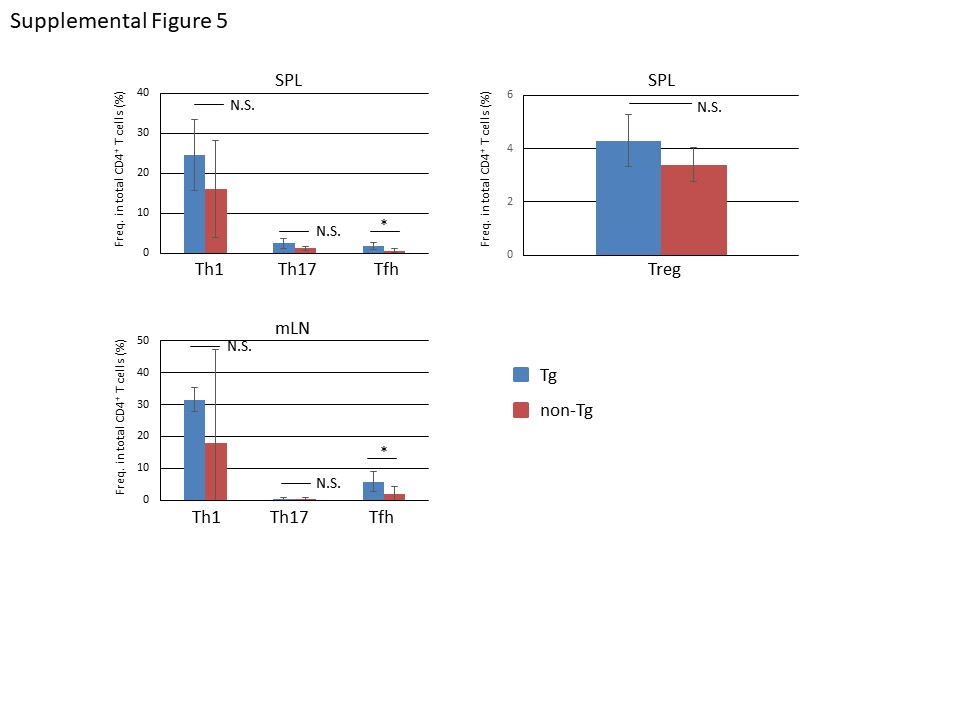

Supplement: Figure S5 — Subpopulation in CD4+ T cells. Spleen and mesenteric lymph node in NOG-pRORγt-γc Tg and NOG-non-Tg mice were analyzed at 16weeks after hematopoietic stem cell transplantation (n = 4). Th1 cells, Th17, or Tfh cells were defined as CD3+CD4+CD45RA−CXCR5−CXCR3+CCR6−, CD3+CD4+CD45RA−CXCR5−CXCR3−CCR6+, or CD3+CD4+CD45RA−CXCR5− cells. Human regulatory T cells were defined as FOXP3+CCR4+CD25+CD4+ T cells. For immunophenotyping, following antibodies were used for staining and analyzed by a BD LSR Fortessa X-20 cell analyzer (BD Biosciences). Anti-CCR7-Brilliant Violet 421, anti-CD45-BV510, anti-CXCR3-APC, anti-CD4-APC, anti-CD45RA-APCCy7, anti-CCR6-PE, anti-CD4-PECy7, anti-CCR4-PECy7, and antimouse CD45-PerCP-Cy5.5 were from BioLegend. Anti-CXCR5-Brilliant Blue 515, anti-CD25-BB515, and anti-CD3-Brilliant Ultraviolet 737 were from BD Biosciences. Dead cells were excluded by 7-AAD (Beckman Coulter). Intracellular staining of FOXP3 was conducted using Anti-Human Foxp3 staining Set phycoerythrin from eBioscience according to the manufacturer’s instruction. Student’s t-test was performed to assess statistical significance (*p < 0.05). [file image_5.jpeg]

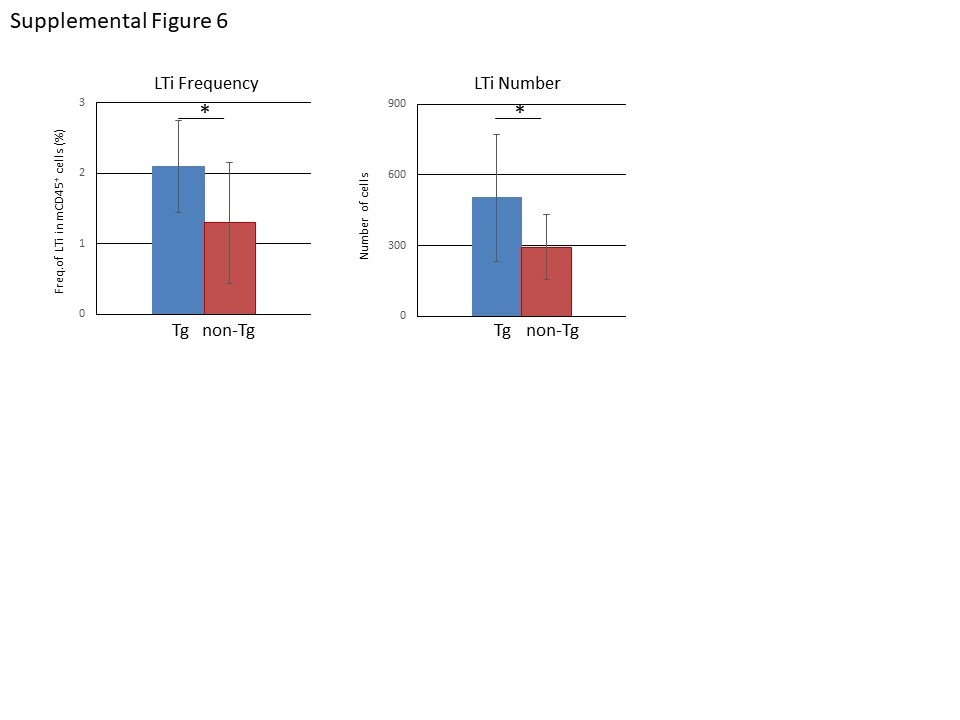

Supplement: Figure S6 — Increase of LTi cells in NOG-pRORγt-γc Tg. The presence of LTi was examined in embryo of NOG-pRORγt-γc Tg and NOG-non-Tg mice at E15. The fetal intestine was smashed with a pestle and mononuclear cells were stained with a cocktail of antibodies; antimouse CD3-FITC, antimouse CD4-PE, antimouse B220, antimouse CD127 (IL-7Rα)-APC, and antimouse CD45-APC-Cy7. LTi cells were defined as CD4+CD127+ cells in CD45+CD3−B220− cells. A part of embryo was used for genotyping by PCR. Mean ± SD from NOG-pRORγt-γc Tg (n = 12) and NOG non-Tg mice (n = 14). Student’s t-test was performed to assess statistical significance (*p < 0.05). [file image_6.jpeg]
